# Automated IS*6110*-based fingerprinting of *Mycobacterium tuberculosis*: Reaching unprecedented discriminatory power and versatility

**DOI:** 10.1371/journal.pone.0197913

**Published:** 2018-06-01

**Authors:** Naira Dekhil, Mohamed Amine Skhairia, Besma Mhenni, Saloua Ben Fradj, Rob Warren, Helmi Mardassi

**Affiliations:** 1 Unit of Typing & Genetics of Mycobacteria, Laboratory of Molecular Microbiology, Vaccinology, and Biotechnology Development, Institut Pasteur de Tunis, Université de Tunis El Manar, Tunis, Tunisia; 2 DST/NRF Centre of Excellence for Biomedical Tuberculosis Research, SAMRC Centre for Tuberculosis Research, Division of Molecular Biology and Human Genetics, Faculty of Medicine and Health Sciences, Stellenbosch University, Cape Town, South Africa; St Petersburg Pasteur Institute, RUSSIAN FEDERATION

## Abstract

**Background:**

Several technical hurdles and limitations have restricted the use of IS*6110* restriction fragment length polymorphism (IS*6110* RFLP), the most effective typing method for detecting recent tuberculosis (TB) transmission events. This has prompted us to conceive an alternative modality, IS*6110*-5’3’FP, a plasmid-based cloning approach coupled to a single PCR amplification of differentially labeled 5’ and 3’ IS*6110* polymorphic ends and their automated fractionation on a capillary sequencer. The potential of IS*6110*-5’3’FP to be used as an alternative to IS*6110* RFLP has been previously demonstrated, yet further technical improvements are still required for optimal discriminatory power and versatility.

**Objectives:**

Here we introduced critical amendments to the original IS*6110*-5’3’FP protocol and compared its performance to that of 24-loci multiple interspersed repetitive unit-variable number tandem repeats (MIRU-VNTR), the current standard method for TB transmission analyses.

**Methods:**

IS*6110*-5’3’FP protocol modifications involved: (i) the generation of smaller-sized polymorphic fragments for efficient cloning and PCR amplification, (ii) omission of the plasmid amplification step in *E*. *coli* for shorter turnaround times, (iii) the use of more stable fluorophores for increased sensitivity, (iv) automated subtraction of background fluorescent signals, and (v) the automated conversion of fluorescent peaks into binary data.

**Results:**

In doing so, the overall turnaround time of IS*6110*-5’3’FP was reduced to 4 hours. The new protocol allowed detecting almost all 5’ and 3’ IS*6110* polymorphic fragments of any given strain, including IS*6110* high-copy number Beijing strains. IS*6110*-5’3’FP proved much more discriminative than 24-loci MIRU-VNTR, particularly with strains of the *M*. *tuberculosis* lineage 4.

**Conclusions:**

The IS*6110*-5’3’FP protocol described herein reached the optimal discriminatory potential of IS*6110* fingerprinting and proved more accurate than 24-loci MIRU-VNTR in estimating recent TB transmission. The method, which is highly cost-effective, was rendered versatile enough to prompt its evaluation as an automatized solution for a TB integrated molecular surveillance.

## Introduction

Tuberculosis (TB), though a curable disease, still represents a major global threat, notably because of the emergence and spread, at an alarming rate, of difficult-to-treat drug-resistant forms. In 2014, 9.6 million new TB cases and 1.5 million deaths were recorded, 480 000 of whom developed multidrug-resistant TB (MDR-TB), a TB form that is resistant to at least isoniazid and rifampicin, the two most effective front-line anti-tubercular drugs [1].

Rapid detection of drug-resistant TB and implementation of an effective system to monitor its spread is a great challenge for any national TB program (NTP) [2]. Ideally, to set effective control measures aimed at reducing the spread of drug-resistant TB, NTPs must be guided by a clear picture of TB transmission dynamics and epidemiology, particularly in those regions where drug resistance is prevalent. Molecular fingerprinting of *M*. *tuberculosis* could address this challenge but still awaits versatile, medium- to high-throughput, and cost-effective typing approach that reflects the true transmission picture.

IS*6110* restriction fragment length polymorphism (IS*6110* RFLP), a typing approach based on the polymorphism generated by the insertion sequence IS*6110*, has proved highly discriminative and has served as the gold standard for the detection and surveillance of outbreaks, notably those involving MDR-TB strains, for many years [3–5]. However, as a hybridization-based approach, IS*6110* RFLP is labor-intensive, time-consuming, refractory to automating, and hence not amenable to high-throughput analyses. The method also suffers the major drawback of not being suitable for data banking and inter-laboratory comparisons [6].

Nowadays, TB molecular surveillance systems rely mainly on multiple interspersed repetitive unit-variable number tandem repeats (MIRU-VNTR) typing for tracing outbreaks and ongoing transmission. Unlike IS*6110* RFLP, MIRU-VNTR is a PCR-based genotyping method whose results are expressed in a digital code, enabling their portability and creation of databases [7]. In its standard 24-locus format, the discriminatory power of MIRU-VNTR was shown to be equal to that of IS*6110* RFLP, except in settings where genetically closely related strains predominate [8]. Compared to whole genome sequencing (WGS), virtually the highest discriminatory typing approach, 24-loci MIRU-VNTR was shown to overestimate recent TB transmission, thus emphasizing the need for an accessible, cost-effective alternative [9,10].

Towards this end, we reconsidered IS*6110*-5’3’FP, a typing approach that we have previously developed [11], and which we believe could represent a valuable alternative to 24-loci MIRU-VNTR. IS*6110*-5’3’FP relies on a single PCR amplification reaction, coupled to simultaneous differential labeling of 5’ and 3’ IS*6110* polymorphic ends, and their automated fractionation on a capillary sequencer. IS*6110*-5’3’FP addresses all the drawbacks of IS*6110* RFLP, while offering increased discriminatory power and flexibility. Indeed, it efficiently and accurately resolves both 5’ and 3’ polymorphic ends on a capillary sequencer, thus enabling inter-laboratory reproducibility, results portability, and hence establishment of databases.

Here we introduced major amendments and refinements to the original IS*6110*-5’3’FP protocol and demonstrated its highest discriminatory power compared to 24-loci MIRU-VNTR. Aside from relying on a single PCR reaction, the overall IS*6110*-5’3’FP protocol was rendered versatile enough to make it a viable alternative to 24-loci MIRU-VNTR with the same benefits in terms of data sharing.

## Materials and methods

### Ethics statement

In this study, no interventions were performed. Only fully anonymized data were processed, and hence no further ethical clearance was required.

### Bacterial strains

The performance of IS*6110*-5’3’FP was assessed using a strain collection of *M*. *tuberculosis* clinical isolates of the Haarlem, LAM, and Beijing genotypes. Those isolates whose IS*6110* RFLP banding pattern is known (some LAM and all Beijing strains) were used to evaluate the ability of IS*6110*-5’3’FP to detect almost all IS*6110* polymorphic fragments. Because IS*6110* RFLP targets only the 3’ polymorphism, the number of peaks expected to be detected by IS*6110*-5’3’FP should be at least twice the number of IS*6110* RFLP bands (5’ and 3’ polymorphic ends). Reproducibility assay was performed with a well characterized IS*6110* RFLP 11-banded clinical strain [12,13], considered herein to as the laboratory reference strain, and which has been previously used in the optimization steps of the original IS*6110*-5’3’FP protocol [11].

Characteristics of the whole strain collection, including the year of isolation, origin, and genotypic data, are provided in S1 Table.

### IS*6110*-5’3’FP modified protocol

**Fig 1:**
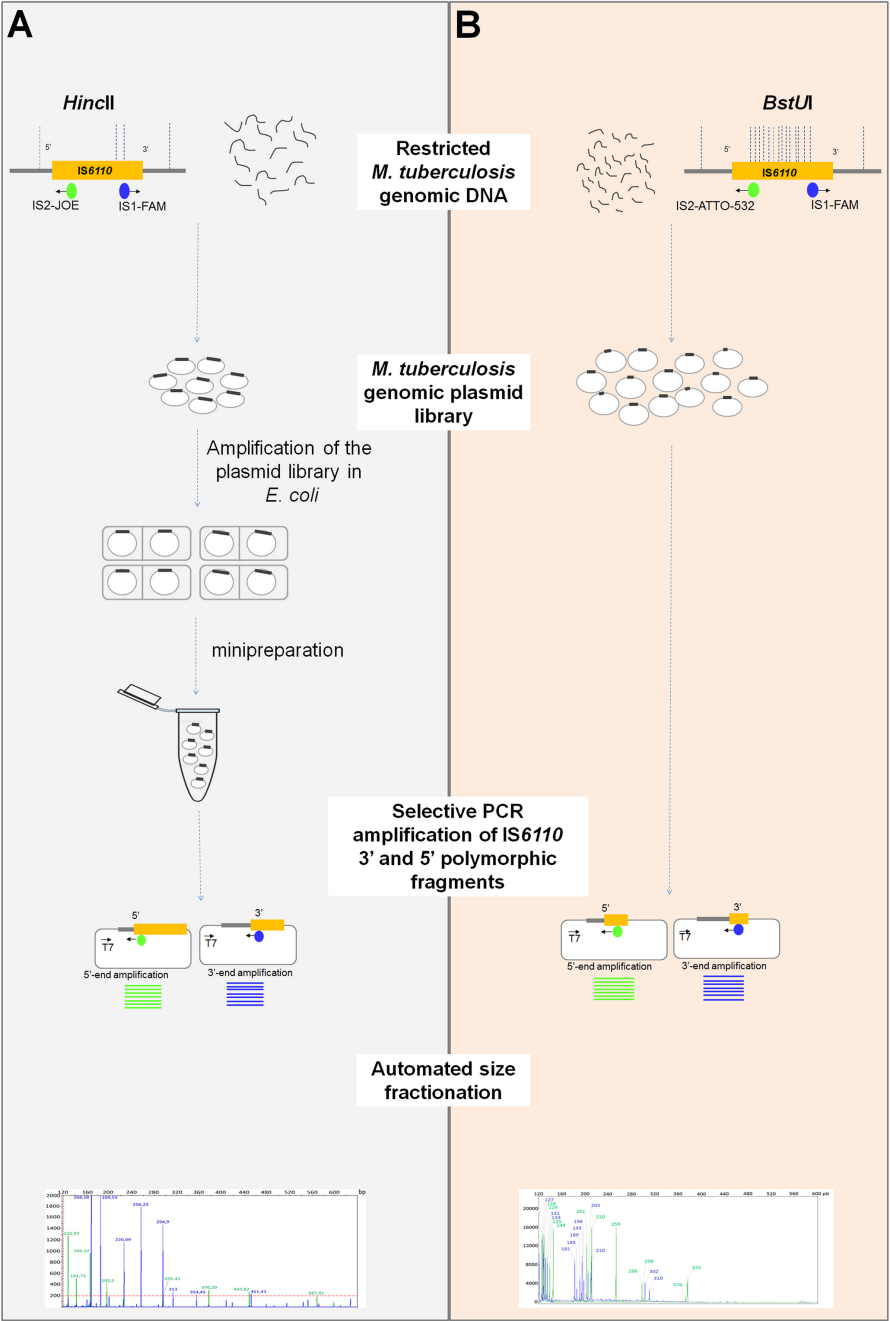
Overview of IS*6110*-5’3’FP original protocol and its simplified highly performing version. (A). The original IS*6110*-5’3’FP protocol as described in Thabet et al. (2014) [11]. (B). The new protocol of IS*6110*-5’3’FP. In this new protocol version, aside from using the frequently cutting *BstU*I enzyme instead of *Hinc*II, there is no need for plasmid library amplification in *E*. *coli*, a modification that considerably shortens the method turnaround. Moreover, amplification in *E*. *coli* could result in the loss of some IS*6110*-containing plasmid most likely because of clone instability. Therefore, omission of this step increases the sensitivity of the method.

The original protocol of IS*6110*-5’3’FP and its amended version are depicted in Fig 1. According to the new protocol, mycobacterial genomic DNA was extracted from a single colony growing on Löwenstein-Jensen medium. Genomic DNA was prepared according to standard recommendations [14]. Next, DNA was fully digested with *BstU*I, a blunt-end restriction enzyme, which cuts several times within the IS*6110* insertion sequence, while leaving intact the 5’ and 3’ ends, and 52 794 times in the chromosomal DNA of the *M*. *tuberculosis* reference strain, H37Rv (NC_000962.3). *BstU*I-Restricted DNA was ligated into *EcoR*V-linearized and dephosporylated plasmid pBS SK+ (Stratagene). Typically, 150 ng of restricted DNA was combined with 50 ng of linearized plasmid vector in a 10-μL ligation reaction. All DNA manipulations including endonuclease restriction and ligation into plasmids were performed according to standard protocols [15]. Restriction endonucleases and other enzymes were used as recommended by the supplier (Amersham Biosciences).

To simultaneously target IS*6110* 3’ and 5’ polymorphic ends, we performed a single PCR reaction involving the T7 primer and the two outward IS*6110*-specific primers IS1 (5’GGCTGAGGTCTCAGATCAG 3’) and IS2 (5’ACCCCATCCTTTCCAAGAAC 3’) [16], fluorescently labeled with FAM and ATTO dyes, respectively. Because cloning is non directional (blunt-end ligation), effective PCR amplification of any given IS*6110* polymorphic fragment takes place only when it is inserted in the right orientation, which basically occurs as frequently as the opposite orientation. Therefore, the use of a single primer in the plasmid (T7 primer, herein) ensures efficient amplification of all polymorphic fragments. Typically, 2 μL of the whole ligation reaction is used as template in the PCR amplification. The latter consisted of 1× *Taq* DNA Polymerase, recombinant buffer (Invitrogen) (4 mM Tris–HCl (pH 8.4), 10 mM KCl, 3 mM MgCl_2_ (Invitrogen), a 1 μM concentration of each primer (Amersham Biosciences), a 50 μM concentration of each deoxynucleotide triphosphate and 1 U of *Taq* DNA Polymerase, recombinant. The reaction was thermal cycled once at 94°C for 5 min, 35 times at 94°C for 1 min, 55°C for 1 min, 72°C for 1 min and then once at 72°C for 10 min. The amplification reaction was performed in a Perkin Elmer GeneAmp PCR system 9700 (Applied Biosystems Inc., CA). As a negative control we performed a PCR reaction using as template DNA the product of a pBS SK+ plasmid auto-ligation reaction.

### Automated fragment size fractionation of IS*6110*-5’3’FP-generated products

A 0.5-μL volume of IS*6110*-5’3’FP PCR product was mixed with 0.5 μl of Gene Scan 600 Liz and 9 μl of Hi-Di Formamide. The mix was denatured for 2 minutes at 95°C. Automated fragment analysis was performed using Liz 600 as a DNA size marker on an ABI PRISM 3100 capillary DNA sequencer (Applied Biosystems Inc., CA, USA). The electrophoresis was done using POP-7 Polymer and a 50-cm length capillary. Electrophoresis parameters were set according to the 3130_50cm_POP-7_GS600LIZ run module (Applied Biosystems Inc., CA, USA). In each run, an equivalent volume of the auto-ligation reaction was included in order to automatically subtract nonspecific fluorescent peaks (S1 Fig).

### Reproducibility study

To assess the reproducibility of IS*6110*-5’3’FP, PCR amplifications were performed on six independent ligation products using the genomic DNA isolated from the 11-banded *M*. *tuberculosis* laboratory reference strain. PCR products were then used in separate capillary runs to test for the reproducibility of the signals in terms of both size and intensity.

### Data analysis

Chromatographs showing FAM- and ATTO 532-fluorescing fragments were imported to GeneMapper software V5 (Applied Biosystems). Fluorescent peak data, which were blinded with regard to IS*6110* copy number, were analyzed using AFLP default and the advanced mode with selection of a starting point of electrophoresis at 3250 data point. Fluorescent peaks whose RFU> 500 were considered. Background signals from the auto-ligation reaction were automatically subtracted from the chromatographs of typed strains, a new modification which simplifies data analysis, since in the original protocol checkerboard dilution testing was performed to determine the threshold for assigning a specific peak. The size of fragments was determined using GeneScan 600Liz version 2 (Applied Biosystems) with a sizing quality up than 0.95, and verified using the plot determined by the software. GeneMapper also recorded the fragment data in a binary format in Excel files which were exported into BioNumerics v6.1, visualized as virtual electrophoresis gels and analyzed. Two IS*6110*-5’3’FP profiles were considered to be different if they had at least one peak difference. Two peaks with a size difference less than 1 bp were considered identical.

### Calculation of the discrimination power

The Hunter-Gaston discriminatory index (HGDI) [17] was used to calculate the discriminatory power of IS*6110*-5’3’FP and 24-loci MIRU-VNTR.

### Clustering analysis

IS*6110*-5’3’FP and 24-loci MIRU-VNTR data were analyzed with the BioNumerics software 6.6 (Applied Maths, East Flanders, BE) in order to construct the similarity matrices and the dendrogram (unweighted pair-grouping method analysis algorithm—UPGMA).

### Cost estimation of IS*6110*-5’3’FP

We carried out an estimation of the total cost of IS*6110-*5’3’FP, from DNA extraction to data acquisition, by taking into account direct costs (e.g., reagents and consumables) and depreciation costs (15%) for those equipments that were directly related to the method and purchased within the last 5 years. This included mainly the automated capillary sequencer used for polymorphic fragments fractionation. In our estimation, labor costs and maintenance fees were included.

### Statistical analyses

Means and standard deviation values were calculated using the data analysis package included within Microsoft Excel 2010 software (Microsoft Corporation, Redmond, WA).

## Results and discussion

The advent of WGS of *M*. *tuberculosis* and its application in molecular epidemiology has revolutionized our understanding of TB transmission dynamics [8,18]. The ability to distinguish *M*. *tuberculosis* isolates differing by a single nucleotide confers to WGS the highest possible level of discrimination and makes it the most valuable tool to investigate tuberculosis outbreaks, uncover unknown transmission events, disclose super spreaders, and refute or confirm epidemiologically suspected transmission links [10,19–22]. WGS has also revealed the limits of 24-loci MIRU-VNTR, the current gold-standard method, in TB transmission analyses. Indeed, WGS showed that 24-loci MIRU-VNTR overestimated recent transmission events [9].

Ideally WGS must be integrated as the typing method of choice into TB surveillance [19], but much has yet to be done to make it of routine use, notably with regard to data analysis, cost-effectiveness, versatility, etc. It may take several years or decades to tackle these obstacles, thus stressing the need for an alternative modality to 24-loci MIRU-VNTR with higher discriminatory power that is cost-effective, amenable to routine high throughput use, and less technically demanding than WGS. For this purpose, we reprised a previously described IS*6110*-based automated typing modality, IS*6110*-5’3’FP, and significantly improved its protocol to meet the above requirements. IS*6110*-5’3’FP was rendered versatile enough to justify its implementation as a medium- to high-throughput alternative to 24-loci MIRU-VNTR, with a more reliable estimation of recent TB transmission rates.

**Fig 2:**
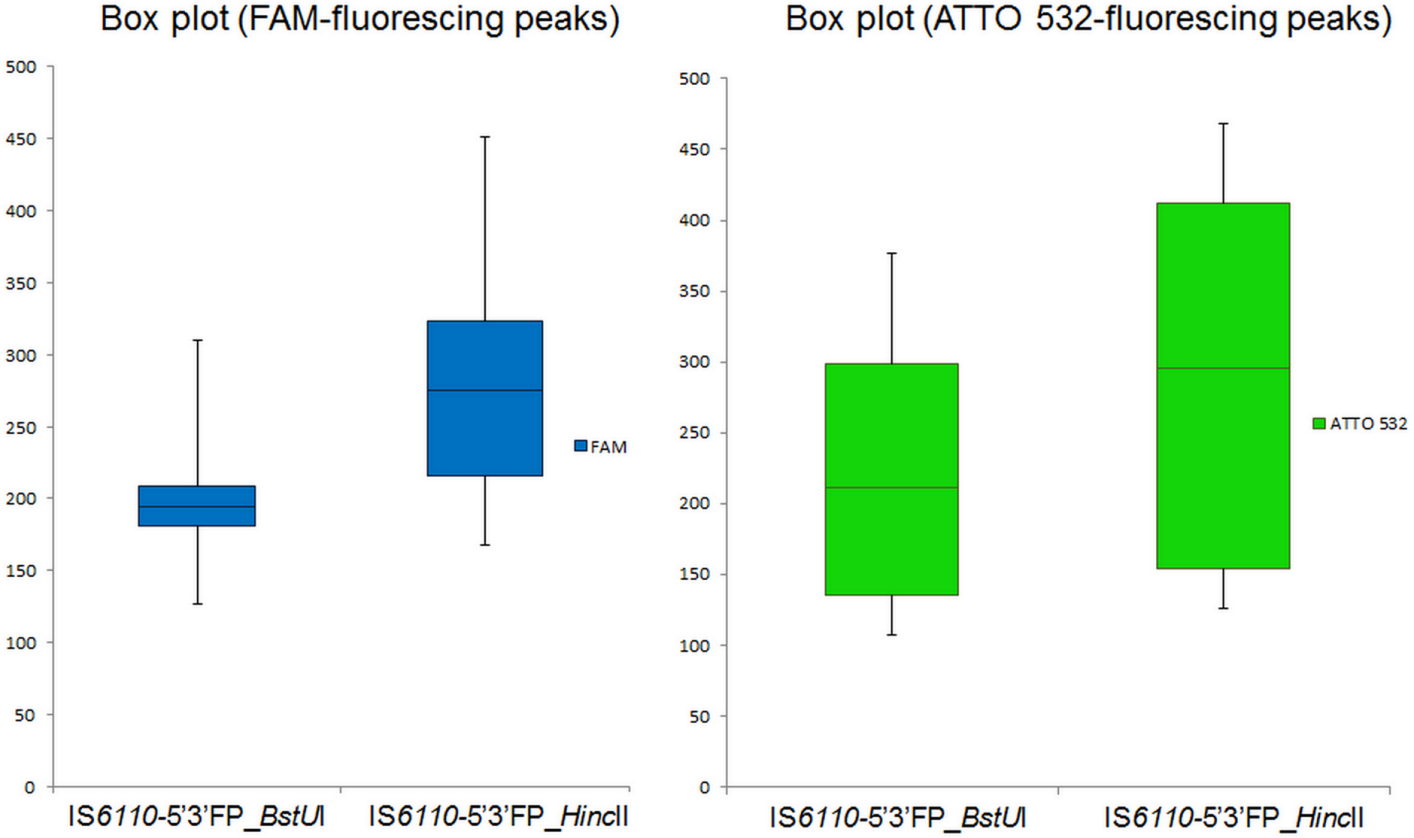
Box plot showing the sizes of IS*6110* polymorphic amplicons generated by IS*6110*-5’3’FP using the 11-banded laboratory reference strain genomic DNA digested either by *BstU*I or *Hinc*II. The IS*6110*-5’3’FP products were fractionated without being diluted on an ABI PRISM 3100 capillary DNA sequencer (Applied Biosystems Inc., CA, USA). The boxes show the 25% to 75% interquartile range.

**Fig 3:**
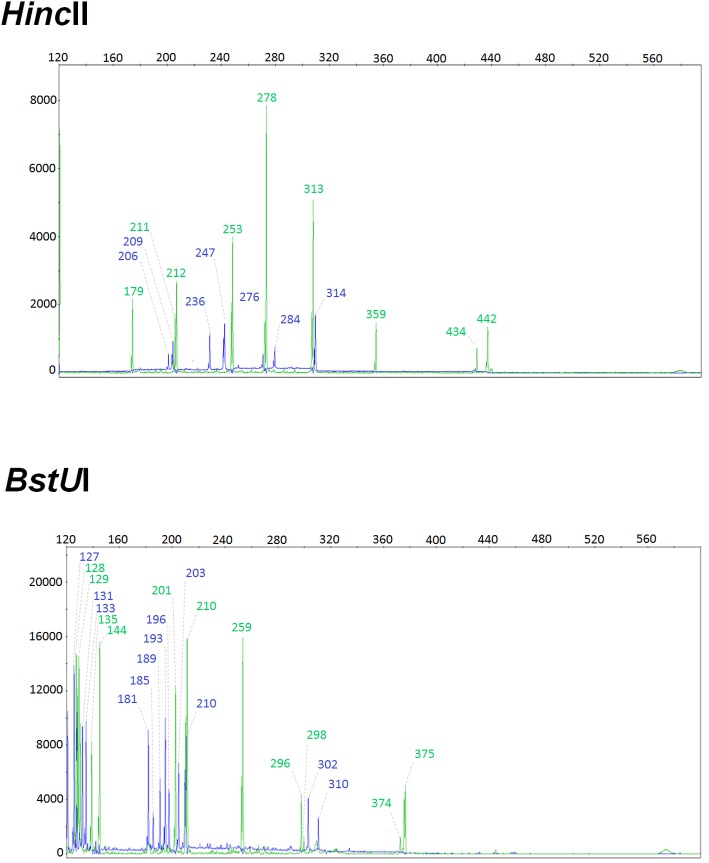
IS*6110*-5’3’FP chromatograms of the 11-banded laboratory reference strain using the original (*Hinc*II-based) and the optimized version developed herein (*BstU*I-based). x-axis = fragments size in base pairs (bp); y-axis = fluorescence intensity in relative fluorescence units (RFU).

The most critical amendment introduced to the original IS*6110*-5’3’FP protocol consisted in the generation of smaller-sized polymorphic fragments in order to increase the efficiency of the cloning and PCR amplification steps (Fig 1). Our objective was to detect and resolve the maximum number of IS*6110* 5’ and 3’ polymorphic fragments, expected to be at least twice the number of IS*6110* RFLP bands. For this purpose, we used the restriction enzyme *BstU*I, which cuts the chromosomal DNA much more frequently than did *Hinc*II (52 794 times vs 7 445 times), thus yielding shorter DNA fragments. As shown in Fig 2, the mean size of IS*6110*-5’3’FP-generated fragments for the 11-banded outbreak strain is 197.13 bp with *BstU*I compared to 295.17 bp when *Hinc*II was used. Consequently, the number of polymorphic peaks generated by IS*6110*-5’3’FP was considerably much higher with *BstU*I (N = 23) than with *Hinc*II (N = 16) (Fig 3). Strikingly, in all tested strains, irrespective of their genotype, the number of IS*6110*-5’3’FP polymorphic peaks was at least twice the number of IS*6110* RFLP bands (Table 1; S1 Table for peak details). This result indicates that polymorphisms on both sides of IS*6110* copies were efficiently detected using this new protocol.

**Table 1:** Number of IS*6110*-5’3’FP-generated peaks relative to IS*6110* copies as determined from IS*6110* RFLP profiles.

Strain	N° of IS*6110*-RFLP bands	N° of IS*6110*-5'3'FP peaks	Genotype
L18	9	20	LAM
L19	10	22	LAM
L20	10	22	LAM
L21	10	22	LAM
L22	11	24	LAM
L23	12	26	LAM
L24	12	28	LAM
L25	12	24	LAM
L18	13	26	LAM
L19	13	26	LAM
B21	19	38	Beijing
B22	19	38	Beijing
B23	19	38	Beijing
B24	19	38	Beijing
B25	19	38	Beijing
B26	19	38	Beijing
B27	19	38	Beijing
B28	19	38	Beijing
B29	19	38	Beijing
B30	19	38	Beijing
B31	19	38	Beijing
B32	19	38	Beijing
B33	19	38	Beijing
B34	19	38	Beijing
B35	19	38	Beijing
B36	19	38	Beijing
B37	19	38	Beijing
B38	19	38	Beijing
B39	19	38	Beijing
B40	19	38	Beijing
B41	19	38	Beijing
B42	19	38	Beijing
B09	21	42	Beijing
B10	21	42	Beijing
B11	21	42	Beijing
B12	21	42	Beijing
B13	21	42	Beijing
B14	21	42	Beijing
B15	21	42	Beijing
B16	21	42	Beijing
B17	21	42	Beijing
B18	21	42	Beijing
B19	21	42	Beijing
B20	21	42	Beijing
B03	22	44	Beijing
B04	22	44	Beijing
B05	22	44	Beijing
B06	22	44	Beijing
B01	23	46	Beijing
B02	23	46	Beijing
B07	23	46	Beijing
B08	23	46	Beijing

LAM, Latin American-Mediterranean lineage.

Aside from using a frequently cutting enzyme, the use of more stable fluorophores could have significantly contributed to the increased sensitivity of IS*6110*-5’3’FP in terms of fragments detection. In its original version, we noticed that 5’-end fragments were much less frequently detected than 3’-end fragments (34.3% vs 65.7%), a finding that was attributed to the relative instability of JOE dye [11]. Here we replaced JOE with ATTO 532, a fluorophore with higher stability and better spectral properties. In doing so, the detection frequency of 5’ polymorphic ends has increased to 47.3%. Furthermore, the ability of IS*6110*-5’3’FP to detect almost all polymorphic fragments also resides in the high resolution power of capillary fractionation, as well as efficient detection of 5’ and 3’ polymorphic fragments of identical size because of their differential labeling. The finding that in 13% of tested strains, IS*6110*-5’3’FP polymorphic peaks exceeded twice the number of IS*6110* RFLP bands (Table 1), was attributed to the lower resolution power of the latter technique, which relies on DNA fragment separation in agarose gels.

**Fig 4:**
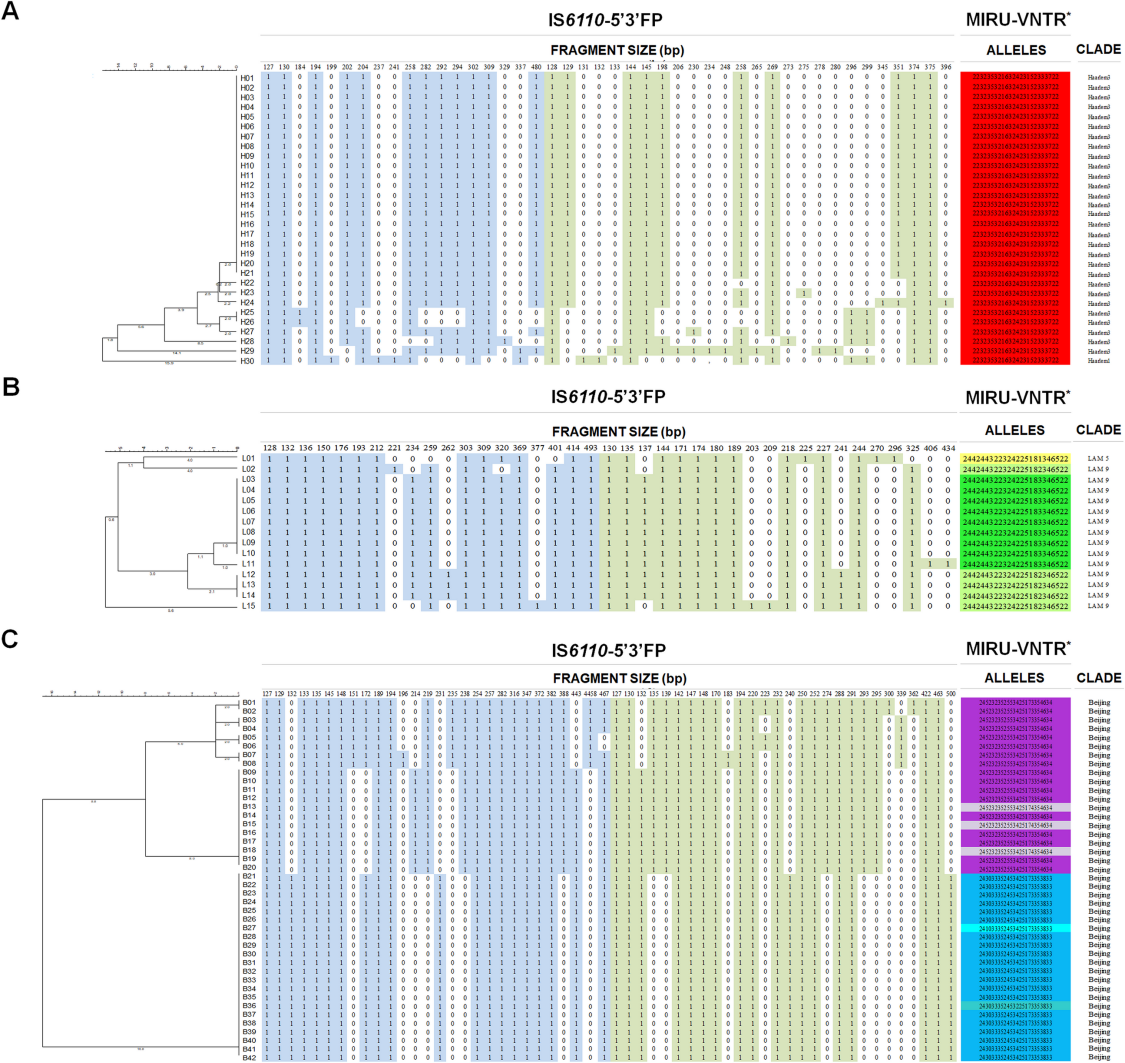
Assessment of the discriminatory power of IS*6110*-5’3’FP and 24-loci MIRU-VNTR. *M*. *tuberculosis* strain collections belonging to Haarlem (A), LAM (B), and Beijing (C) genotypes were used.

With these modifications, IS*6110*-5’3’FP was found much more discriminative than 24-loci MIRU-VNTR, particularly with epidemiologically linked *M*. *tuberculosis* Haarlem and LAM genotype isolates prevailing in a geographically-confined region of Northern Tunisia. Indeed, IS*6110*-5’3’FP subdivided a 24-loci MIRU-VNTR-defined cluster (HGDI = 0.000), involving 30 *M*. *tuberculosis* Haarlem isolates, into one major (21 isolates) and one minor (2 strains) subclusters, and 7 unique profiles, resulting in a HGDI of 0.514 (Fig 4A, Table 2). Likewise, IS*6110*-5’3’FP significantly reduced the overall percentage of LAM clustered isolates compared to 24-loci MIRU-VNTR (HGDI: 0.704 vs 0.561, respectively) (Fig 4B, Table 2). The higher discriminatory ability of IS*6110*-5’3’FP compared to 24-loci MIRU-VNTR was further demonstrated with lineage 2 Beijing genotype *M*. *tuberculosis* isolates (N = 42) from South Africa (HGDI: 0.650 vs 0.617) (Fig 4C, Table 2). Previous studies conducted in different geographical regions involving large *M*. *tuberculosis* strain collections have proved that 24-loci MIRU-VNTR typing has a discriminatory power equal, or slightly higher to IS*6110*-RFLP, particularly when combined with spoligotyping [23–26]. Here, by detecting and efficiently resolving both 5’ and 3’ IS*6110* polymorphic ends, IS*6110*-5’3’FP proved its higher discriminatory power with the three major *M*. *tuberculosis* genotypes. However, the performance of IS*6110*-5’3’FP must be assessed in various geographical areas in which neither IS*6110* RFLP nor 24-loci-MIRU VNTR were sufficient enough to fully discriminate Beijing or non-Beijing strain families [24,27]. On the other hand, the discriminatory ability of IS*6110*-5’3’FP has to be evaluated for strains with low-copy number of IS*6110* (five or fewer). However, since IS*6110*-5’3’FP simultaneously detects both 5’ and 3’ polymorphisms, one can rightfully anticipate its ability to efficiently discriminate *M*. *tuberculosis* isolates with few IS*6110* copies (< 6 IS*6110* RFLP bands). Indeed, the combined use of 5’-end and 3’-end IS*6110* probes has previously been shown to significantly increase the discrimination power of IS*6110* RFLP [28]. However, it should be noted that like all IS*6110*-based typing techniques, IS*6110*-5’3’FP is with no value for strains lacking the IS*6110* element. In such circumstances, MIRU-VNTR remains the most indicated approach.

**Table 2:** Comparison of the discriminatory power of IS*6110*-5’3’FP and 24-loci MIRU-VNTR.

	24 Loci MIRU-VNTR			IS*6110*-5’3’FP		
	Haarlem	LAM	Beijing	Haarlem	LAM	Beijing
	(N = 30)	(N = 15)	(N = 42)	(N = 30)	(N = 15)	(N = 42)
**HGDI**	0.000	0.561	0.617	0.514	0.704	0.650
**No. of unique types (%)**	0 (0)	1 (6.6)	2 (4.7)	7 (23.3)	4 (26.6)	0 (0)
**No. of clusters**	1	2	3	2	2	6
**No. of clustered isolates (%)**	30 (100)	14 (93.3)	40 (95.2)	23 (76.6)	11 (73.3)	42 (100)
**Maximum no. of isolates in a cluster**	30	9	20	21	8	22

HGDI, Hunter-Gaston discriminatory index; LAM, Latin American-Mediterranean lineage; MIRU-VNTR, Multiple Interspersed Repetitive Unit-Variable Number Tandem Repeats

More importantly, detection of both IS*6110* 5’ and 3’ polymorphisms makes IS*6110*-5’3’FP more reliable than IS*6110* RFLP in studying the clonal relationship of *M*. *tuberculosis* isolates. Since matching IS*6110* RFLP fingerprint pattern generated by the IS*6110* 3’-end probe (used in the standard IS*6110* RFLP protocol) does not always indicate the same IS*6110* insertional event, it has been suggested that a combination of IS*6110* 3’ and 5’ probes should increase the reliability of IS*6110* fingerprinting in detecting clonally-related strains [28]. The reliability of IS*6110*-5’3’FP in identifying clonally related strains is further enhanced by the accurate resolution of polymorphic fragments in a capillary sequencer, as well as efficient differentiation of 5’ and 3’ polymorphic fragments of identical size. Therefore, not only is IS*6110*-5’3’FP highly discriminatory, it is likely to be epidemiologically more informative, a hallmark which further reinforces its use in population-based studies.

IS*6110*-5’3’FP protocol was considerably improved in terms of technical flexibility and work flow, offering several advantages over existing gold-standard methods. Firstly, the overall turnaround time of the technique was considerably reduced to 4 hours, as we omitted the amplification step of the ligation product in *E*. *coli* (Fig 1). In doing so, not only did we significantly reduce the turnaround time, but we also improved the ability of the method to detect increased number of polymorphic fragments. For more flexibility, the plasmid ligation step can be readily achieved using ready-to-use blunt-end cloning kits. In comparison to other ligation-mediated PCR approaches, cloning in a pre-linearized plasmid vector is much more simple and efficient than ligation to adapters or linkers [29–31].

Unlike 24-loci MIRU-VNTR, which necessitates several PCR reactions and optimizations (for particular loci), IS*6110*-5’3’FP relies on a single PCR reaction, a characteristic which makes it more amenable to high-throughput analyses.

The use of a capillary sequencer to fractionate fluorescently labeled 5’ and 3’ polymorphic fragments confers to IS*6110*-5’3’FP the same potential for data portability as 24-loci MIRU-VNTR, yet, sizing is likely to be much more accurate for the former approach. Indeed, the small size of *BstU*I-generated polymorphic fragments (<500 bp), does not suffer sizing inaccuracies such as those encountered with long PCR fragments (>750 bp) obtained with some MIRU-VNTR loci [32–35]. Furthermore, small-sized DNA fragments are much less vulnerable to run-to-run sizing variation in capillary electrophoresis systems. In this respect, it is worthy of mentioning that IS*6110*-5’3’FP proved highly reproducible as the run-to-run standard deviation of fragment size estimates did not exceed 0.67 bp (S2 Table). Since this reproducibility assay was performed using IS*6110*-5’3’FP products of six independent reactions, each with a new DNA preparation, we anticipate for a good inter-laboratory reproducibility of IS*6110*-5’3’FP.

Strikingly, IS*6110*-5’3’FP proved much more cost-effective than 24-loci MIRU-VNTR (12,56 vs 53,6 USD per strain) (Table 3) [35]. This low cost compared to 24-loci MIRU-VNTR stems mainly in the fact that IS*6110*-5’3’FP relies on a single PCR reaction. At best, 24-loci MIRU-VNTR can be performed in eight triplex PCR reactions, but still remains costly, particularly when commercially available kits are used to ensure reliable PCR amplifications. Furthermore, while IS*6110*-5’3’FP can genotype as much as 95 *M*. *tuberculosis* clinical strains in a single capillary electrophoresis run (plus one auto-ligation control of a 96-well microplate), 24-loci-MIRU VNTR could process only 4 (24 simplex PCR reactions) to 12 strains (8 triplex PCR reactions). With regard to WGS, and considering the current costs of ~ 60 USD per genome (for a batch of at least 300 genomes) (Table 3), IS*6110*-5’3’FP is clearly much less expensive. Yet, despite the continuous declining costs of WGS, the use of IS*6110*-5’3’FP as an automatized solution for a TB integrated molecular surveillance could still be justified, since analysis of fluorescent fragment length is much easier and much less demanding in terms of bioinformatics skills and IT infrastructure.

**Table 3:** Comparative cost estimates (USD).

	IS*6110*-5’3’FP	24-loci MIRU-VNTR^a^	WGS^b^
**Unit cost**			
Polymorphic fragments generation steps	8,96	31,00	-
Automated fragment analysis	3,60	22,60	-
**Total unit cost**	12,56	53,6	60,00

WGS, whole genome sequencing; 24-loci MIRU-VNTR, 24-loci multiple interspersed repetitive unit-variable number tandem repeats.

^a^1 reaction involves PCR amplification of the 24 loci for each isolate [35].

^b^Illumina MiSeq, 2x300 paired ends for more than 300 assays.

## Conclusions

Numerous previous studies have highlighted the benefit of complementing the classical contact tracing strategy with molecular typing data to get a clear picture of TB transmission and, hence, effective surveillance of outbreaks, particularly those involving MDR-TB strains. One of the most limiting factors to achieve such a goal consists in the absence of a typing method that reliably estimate TB transmission, while offering sufficient technical flexibility to be routinely used in large-scale population-based studies. Aside from being highly cost-effective, the automated IS*6110*-based typing protocol developed herein showed an unprecedented discriminatory power and versatility, and could thus represent a step forward for the effective implementation of a TB integrated molecular surveillance. Indeed, the new IS*6110*-5’3’FP protocol, which is executable in a few hours, could be fully automated and could even gain more in terms of flexibility with the use of technically simple and cost-effective new capillary electrophoresis technologies, such as QiaXcell, Bioanalyzer, etc. Importantly, given the small size of IS*6110*-5’3’FP-generated polymorphic fragments, the method does not suffer sizing inaccuracies as reported for some MIRU-VNTR loci. With the optimized protocol described in this study, IS*6110*-5’3’FP proved robust enough to undergo performance and inter-laboratory reproducibility assessment.

## Supporting information

S1 Fig Typical chromatogram of IS*6110*-5’3’FP background signal. IS*6110*-5’3’FP was performed using the auto-ligation product of pBS SK+ plasmid vector.
(TIF)

S1 Table *M*. *tuberculosis* strain collections of Haarlem, LAM, and Beijing genotypes used to assess the performance of IS*6110*-5’3’FP new protocol. Genotypic data, including details on the fluorescent peaks generated by IS*6110*-5’3’FP, are included.
(XLSX)

S2 Table Results of the reproducibility assay. (XLSX)
