# Supplementary figures and images for: Automated IS6110-based fingerprinting of Mycobacterium tuberculosis: Reaching unprecedented discriminatory power and versatility
Source: PLoS One. 2018 Jun 1;13(6):e0197913. doi: 10.1371/journal.pone.0197913 (PMC5983439; doi:10.1371/journal.pone.0197913)

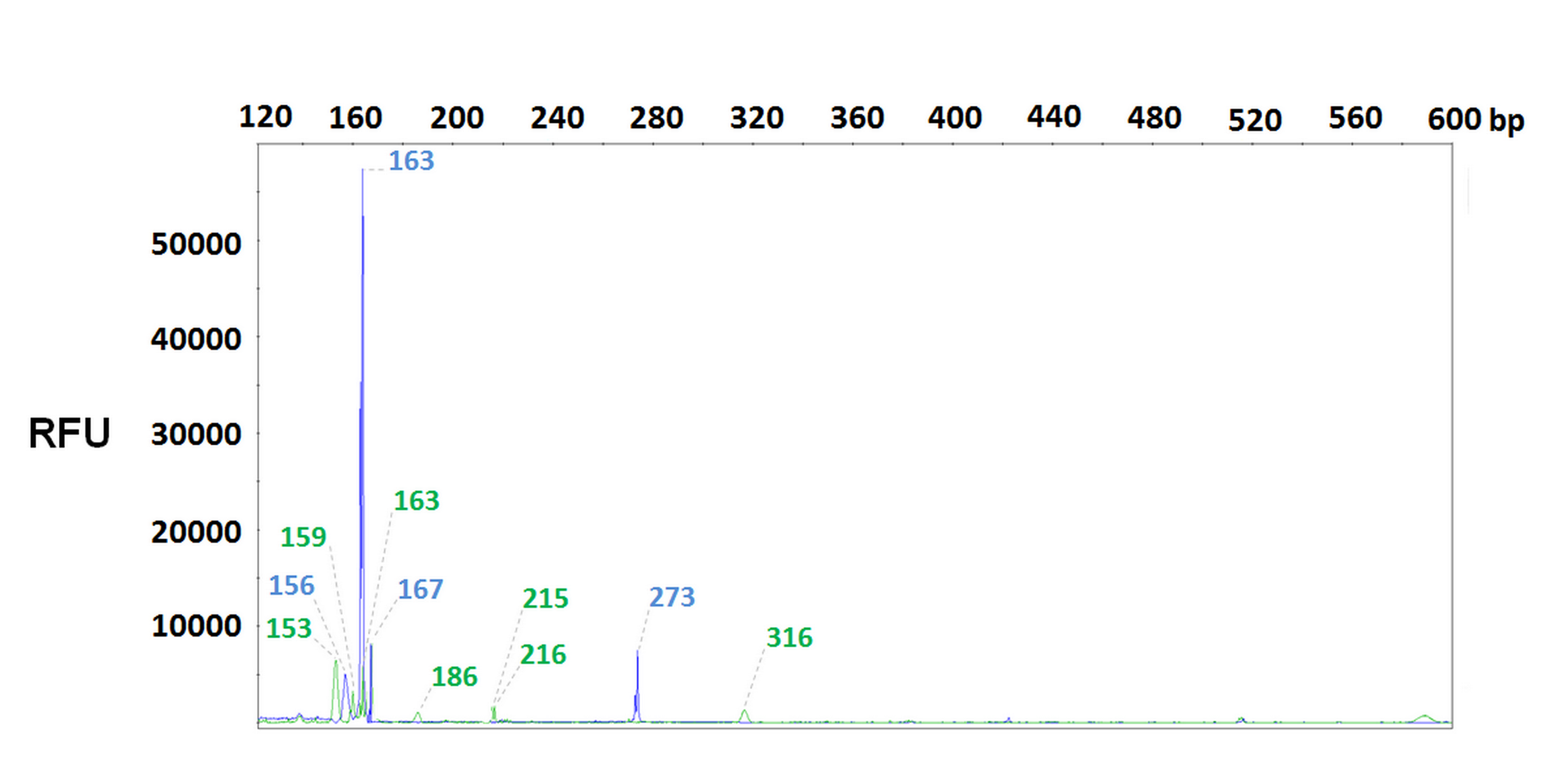

Supplement: S1 Fig — IS6110-5’3’FP was performed using the auto-ligation product of pBS SK+ plasmid vector. (TIF) [file pone.0197913.s001.tif]
